# A Phylogenetic Codon Substitution Model for Antibody Lineages

**DOI:** 10.1534/genetics.116.196303

**Published:** 2017-03-16

**Authors:** Kenneth B. Hoehn, Gerton Lunter, Oliver G. Pybus

**Affiliations:** * Department of Zoology, University of Oxford, OX1 3PS, United Kingdom; † Wellcome Trust Centre for Human Genetics, University of Oxford, OX3 7BN, United Kingdom

**Keywords:** B-cell receptor, antibody, evolution, lineage, phylogenetic tree, Genetics of Immunity

## Abstract

Phylogenetic methods have shown promise in understanding the development of broadly neutralizing antibody lineages (bNAbs). However, the mutational process that generates these lineages, somatic hypermutation, is biased by hotspot motifs which violates important assumptions in most phylogenetic substitution models. Here, we develop a modified GY94-type substitution model that partially accounts for this context dependency while preserving independence of sites during calculation. This model shows a substantially better fit to three well-characterized bNAb lineages than the standard GY94 model. We also demonstrate how our model can be used to test hypotheses concerning the roles of different hotspot and coldspot motifs in the evolution of B-cell lineages. Further, we explore the consequences of the idea that the number of hotspot motifs, and perhaps the mutation rate in general, is expected to decay over time in individual bNAb lineages.

RECENT advances in sequencing technology are giving an unprecedented view into the genetic diversity of the immune system during infection, especially in the context of chronic infections caused by viruses. Broadly neutralizing antibody (bNAb) lineages, which produce B-cell receptors (BCRs) capable of binding a wide range of viral epitopes, are of particular interest (Haynes *et al.* 2012). Within such lineages, all B cells descend from a shared common ancestor and are capable of rapid sequence evolution through the processes of somatic hypermutation (SHM) and clonal selection. For chronically infecting viruses such as HIV-1, this coevolutionary process may continue for years (Wu *et al.* 2015). Because immunoglobulin gene sequences from bNAb lineages undergo rapid molecular evolution, selection, and diversification, they would appear to be suitable for evolutionary and phylogenetic analysis, and these methods have already been applied to various immunological questions such as inferring the intermediate sequences of bNAb lineages (Sok *et al.* 2013; Hoehn *et al.* 2016). Intermediate ancestors of B-cell lineages are of particular interest because they may act as targets for stimulation by vaccines (Haynes *et al.* 2012).

However, the biology of mutation and selection during SHM is different from that which occurs in the germline, and therefore it is unlikely that standard phylogenetic techniques will be directly applicable to studying bNAb lineages without suffering some bias and error. One of the most important assumptions of likelihood-based phylogenetics is that evolutionary changes at different nucleotide or codon sites are statistically independent. Without this assumption, likelihood calculations become computationally impractical as the length and number of sequences increases (Felsenstein 1981). Unfortunately, in contrast to germline mutations, SHM of BCR sequences is driven by a collection of enzymes that cause some sequence motifs (between 2 and 7 bp) to mutate at a higher rate than others (Smith *et al.* 1996; Teng and Papavasiliou 2007; Elhanati *et al.* 2015). This context sensitivity clearly violates the assumption of independent evolution among sites. Furthermore, because hotspot motifs are, by definition, more mutable than nonhotspot motifs, their frequency within a B-cell lineage may decrease over time as they are replaced with more stable motifs (Sheng *et al.* 2016). These changes will not be passed on to subsequent generations through the germline because the mutational process is somatic. This effect may have a number of consequences for molecular evolutionary inference, for example it may render inappropriate the common practice of estimating equilibrium frequencies from the sequences themselves. At present it is unknown how the violation of these assumptions will affect phylogenetic inference of BCR sequences in practice, and the problem of ameliorating such effects remains an open issue.

Some approaches have been developed to study the substitution process in BCR data in the context of biased mutation. Some of these are nonphylogenetic in nature (Hershberg *et al.* 2008; Yaari *et al.* 2012) and focus on the expected number of germline-to-tip replacement mutations in comparison to a null model. Kepler *et al.* (2014) developed a nonlinear regression model approach that, combined with an empirical model of mutation rate at each site, allowed the authors to test for the effects of selection and mutation on BCR genetic diversity. The substitution model detailed in McCoy *et al.* (2015) accounts for biased mutation by comparing values of *ω* inferred from a given data set to those inferred from out-of-frame rearrangements, and focuses on analyzing mostly nonphylogenetically related sequences from an entire BCR repertoire.

However, no existing approaches explicitly parameterize the effect of biased mutation of BCR hotspot motifs within a phylogenetic substitution model, and this is a crucial step in uniting the well-established field of model-based molecular phylogenetics with BCR genetics and immunology. The aim of this article is to develop such a model. Specifically we introduce a model that can partially account for the effect of context-dependent mutability of hot- and coldspot motifs, and explicitly model descent from a known germline sequence. The model we introduce is a modification of the GY94 (Goldman and Yang 1994) codon-substitution model. Although an approximation, our new model can detect and quantify the effect of SHM on BCR sequences whilst preserving the assumption of independence among codon sites to maintain computational feasibility. This model shows a significantly better fit than the standard GY94 model to three previously published and long-lived bNAb lineages in HIV-1 infected patients. Through simulations, we validate the effectiveness of the model, and show its ability to reduce bias in the estimation of other evolutionary parameters such as tree length. Further, we use this model as a framework for testing hypotheses of hotspot motif symmetry and hierarchy of mutability, and we explore its potential applications such as improved ancestral state reconstruction.

## Materials and Methods

### A molecular evolutionary model for antibody lineages

**Figure 1:**
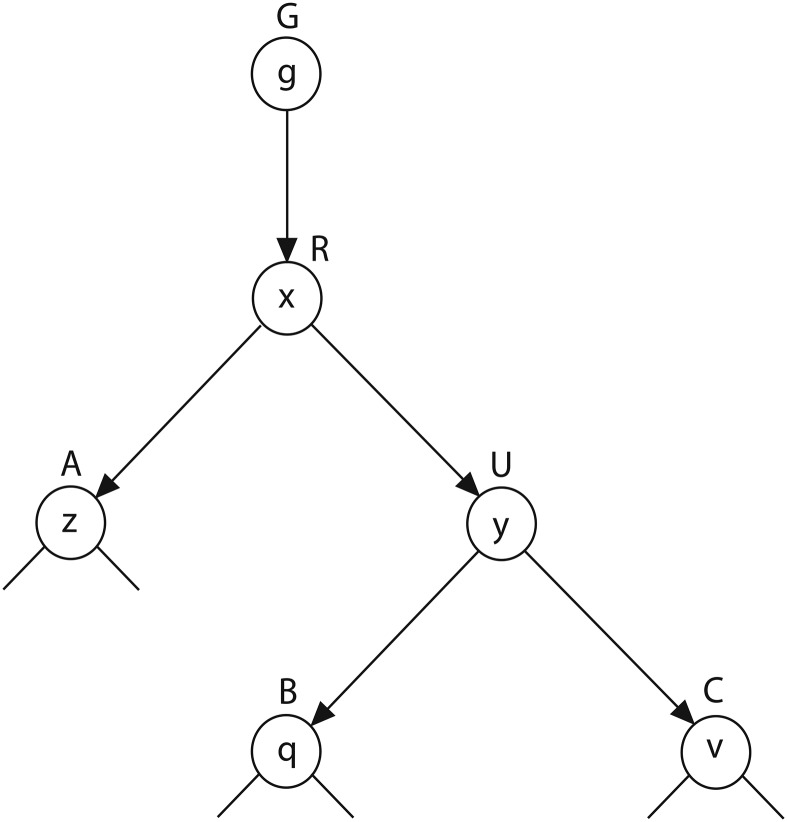
Proposed evolutionary model for antibody lineages. All sequences descend from given germline node *G*, which has sequence *g*. Arrows indicate the direction of evolutionary change. Note that this known ancestor *G* is not necessarily the most recent common ancestor of the lineage, which is node *R* and sequence *x*. See similarities to tree in Boussau and Gouy (2006).

The model we propose differs from most substitution models in some important ways. First, codon substitutions are weighted by the probability that they occurred within a particular hotspot motif, which makes the substitution process nonreversible and has important implications for parameter inference and likelihood calculation. Second, BCR sequences are the direct descendants of a germline ancestor, the sequence of which is assumed to be known or inferred *a priori*. Both these conditions are illustrated graphically in Figure 1.

To represent the molecular evolution of long-lived B-cell lineages more accurately, we develop here a new substitution model that models the effects of motif-specific mutation across BCR sequences. This model, named the HLP17 model, is a modification of the GY94 substitution model (more specifically, it is a modification of the M0 model, because *ω* is kept constant among sites and lineages; Yang *et al.* 2000). Specifically, we add to the GY94 model an additional parameter, *h^a^*, which represents the change in relative substitution rate of a hotspot/coldspot mutation in motif *a*. Explicitly modeling the full context dependence of hotspot motifs would make likelihood calculations computationally infeasible. Instead, we weight *h^a^* by $b_{ij}^{a},$ which is the probability that the mutation from codon *i* to codon *j* was a hotspot mutation in motif *a*, averaged across all possible combinations of codons on the 5′ and 3′ flanks of the target codon. This is a mean-field approximation (*i.e.*, the expected effect is averaged across all possible scenarios) and is similar to the singlet-doublet-triplet model of Whelan and Goldman (2004). A “hotspot mutation” is defined as a mutation occurring within the underlined base of the specified motif [*e.g.*, the trimer motif and its reverse complement WRC/GYW; nucleotides represented using the International Union of Pure and Applied Chemistry (IUPAC) coding scheme with W = A or T, Y = T or C, and R = A or G].

In the HLP17 model, each entry *q_ij_* in the transition rate matrix **Q** is parameterized by:

*π_j_* = Baseline frequency of codon *j*.
*κ* = Transition/transversion mutation relative rate ratio.
*ω* = Nonsynonymous to synonymous mutation relative rate ratio.
*a* = Motif in which mutation rate is modified at underlined base. Here, *a* ∈ {WRC, GYW, WA, TW, SRC, GRS}, but in principle any other motif ≤4-nt long could be used.
*h^a^* = Change in mutability due to mutation in motif *a*; *h^a^* ≥ −1.

and the transition matrix **Q** itself is defined by:

$$q_{\mathrm{ij}}=\{\begin{array}{ll} 0 & \;i\to j\;>1\text{-}\mathrm{nt}\;\mathrm{change}\\ \pi_{j}\left(1+\sum_{a}b_{ij}^{a}h^{a}\right) & i\to j\;\mathrm{synonymous}\;\mathrm{transversion}\\ \kappa \pi_{j}\left(1+\sum_{a}b_{ij}^{a}h^{a}\right) & i\to j\;\mathrm{synonymous}\;\mathrm{transition}\\ \omega \pi_{j}\left(1+\sum_{a}b_{ij}^{a}h^{a}\right) & i\to j\;\mathrm{nonsynonymous}\;\mathrm{transversion}\\ \omega \kappa \pi_{j}\left(1+\sum_{a}b_{ij}^{a}h^{a}\right) & i\to j\;\mathrm{nonsynonymous}\;\mathrm{transition,} \end{array}$$ (1)

where $b_{ij}^{a}$ is the probability that a mutation from *i* to *j* involves the underlined base in motif *a*. The values of $b_{ij}^{a}$ are calculated by marginalizing over all possible 5′- and 3′-flanking sense codons as follows:

$$b_{ij}^{a}=\;\sum_{k=1}^{61}\sum_{m=1}^{61}\pi_{k}\pi_{m}I\left(i,j,k,m,a\right),$$ (2)

where *I* is the indicator function:

$$I\left(i,j,k,m,a\right)=\{\begin{array}{ll} 1 & kim\to kjm\text{ is a mutation from motif }a\\ 0 & \text{otherwise}. \end{array}$$ (3)

This model, though an approximation, has several useful properties. Most importantly, because codon changes are modeled as occurring independently of each other, the phylogenetic likelihood can still be calculated using Felsenstein’s pruning algorithm, which greatly reduces computational time (Felsenstein 1981). This substitution model also has the intuitive property that, if no hotspot motif is specified, then all *h^a^* = 0 and the model simplifies to the GY94 model. Thus the M0 submodel of the GY94 model, when using the same given germline sequence, is a special case of the HLP17 model.

While in standard GY94-type models the vector **π** represents the equilibrium frequencies of codons, this is not the case for the HLP17 model. This can be checked by direct calculation of the total flux in and out of a codon *j*; in general $\sum_{i\neq j}\pi_{i}q_{ij}\neq \sum_{i\neq j}\pi_{j}q_{ji}$ for HLP17 because the matrix $b_{ij}^{a}$ is generally not symmetric in *i* and *j*. Although equilibrium frequencies do exist (and can be calculated numerically), we are in fact interested in the model’s nonequilibrium behavior, since the ancestral sequence is likely to be far from equilibrium, and by the time the codons are observed the sequence is unlikely to have reached equilibrium. As a result, the best-fit values of **π** may even change according to the time at which a B-cell lineage is sampled. Thus the values of **π** in our model are more appropriately interpreted as best-fit constant codon frequencies given the data and other model parameters, and should not be directly interpreted as equilibrium frequencies. More specifically, we use the CF3x4 model as implemented in codonPhyML (Kosakovsky Pond *et al.* 2010; Gil *et al.* 2013) to find the best-fitting codon frequencies. In this model, the frequencies of A, C, G, and T at each of the three codon positions are estimated through ML as 12 additional parameters.

Because the M0 version of the GY94 model, using the same germline root sequence, is a special case of the HLP17 model (*i.e.*, when all *h* parameters = 0), the two models are nested and can be compared using a likelihood-ratio test. Let *L*_max_(HLP17) and *L*_max_(M0) be the maximum likelihoods (MLs) obtained under the HLP17 and M0 models, respectively. The likelihood-ratio statistic 2 log[*L*_max_(HLP17)/*L*_max_(M0)] is then approximately chi-squared distributed with degrees of freedom equal to the number of additional *h* parameters (Huelsenbeck and Rannala 1997). For each bNAb data set, we calculate *L*_max_(HLP17) by cooptimizing *h* and other model parameters, whereas *L*_max_(M0) is calculated by constraining all *h^a^* = 0 while optimizing the other model parameters. Importantly, because HLP17 uses a given germline sequence rather than codon frequencies during likelihood calculations at the root (see *Likelihood calculation and ancestral state reconstruction* below), it is only nested with M0 if M0 also uses the same sequence as its direct ancestor. Using codon frequencies in absence of a known root, as is usually done during likelihood calculations (Felsenstein 1981), would make the likelihood values of these models incomparable through a likelihood-ratio test.

Within this framework, a hierarchical network of hotspot models can be specified by fixing certain values of *h^a^* to zero and by setting some values of *h^a^* to be equal. For instance, a symmetric WRC/GYW model is specified by setting *h*^WR^^C^ = *h*^G^^YW^ and by setting all other values of *h^a^* to zero, leaving just one parameter (*h*^WR^^C^) to be estimated using ML. Pairs of models that are nested (*e.g.*, strand symmetric *vs.* asymmetric motifs) can be formally compared using likelihood-ratio tests; nonnested models may be compared using the Akaike information criterion (AIC).

### Likelihood calculation and ancestral state reconstruction

Our implementation of the HLP17 model requires a nonstandard approach to likelihood calculations. In contrast to most substitution models, the rate parameters in the **Q** matrix of the HLP17 model are not necessarily time reversible, *i.e.*, the model does not necessarily satisfy the detailed balance condition $\pi_{i}q_{ij}=\pi_{j}q_{ji}.$ Time reversibility is useful because it means that likelihood calculations can be undertaken on an unrooted tree, which subsequently can be rooted on any branch. This property, known as the “pulley principle,” only holds for reversible models and helps to speed up search algorithms for ML tree inference (Felsenstein 1981) and marginal ancestral sequence reconstructions (Koshi and Goldstein 1996; Bollback *et al.* 2007).

However, in the case of B-cell lineage evolution it is necessary to root the phylogeny at the germline sequence during parameter estimation. Fortunately, Boussau and Gouy (2006) have introduced an algorithm that uses partial-likelihood values to calculate the likelihood at each node in the tree for nonreversible models. This provides a similar increase in computational efficiency during likelihood evaluation to that provided by the pulley principle in reversible models. The algorithm we use for likelihood calculations is the same as that in Boussau and Gouy (2006), with the exception that we require tip sequences to descend from a known germline sequence (node *G*; Figure 1).

Using effectively the same formulas as in Boussau and Gouy (2006) and Boussau *et al.* (2008), we use two partial-likelihood functions, *L*_low_ and *L*_upp_, which describe the partial likelihoods of the branches above and below a given edge. Given the tree in Figure 1, for site *s* in the sequence alignment:

$$L_{\text{low}}\left(UC,v\right)=\{\begin{array}{ll} 1 & \text{if codon }v\text{ is equal to site }s\text{ in leaf }C\\ 0 & \text{otherwise,} \end{array}$$ (4)

$$L_{\text{low}}\left(RU,y\right)=\sum_{q}P_{yq}L_{\text{low}}\left(UB,q\right)\sum_{v}P_{yv}L_{\text{low}}\left(UC,v\right),$$ (5)

$$L_{\text{upp}}\left(RU,x\right)=P_{gx}\sum_{z}P_{xz}L_{\text{low}}\left(RA,z\right),$$ (6)

$$L_{\text{upp}}\left(UB,y\right)=\sum_{x}P_{xy}L_{\text{upp}}\left(RU,x\right)\sum_{v}P_{yv}L_{\text{low}}\left(UC,v\right),$$ (7)

where *P_gx_* is the probability of a transition from codon *g* to codon *x* along edge *GR*. Importantly, because the character at the root node *G* is given as *g*, equilibrium frequencies are not used at the root node during likelihood calculations. The likelihood for site *s* (*L_s_*) at any edge, for instance *UB*, is then:

$$L_{s,UB}=P\left({Χ}_{s}|G=g\right)=\sum_{y}L_{\text{upp}}\left(UB,y\right)\sum_{q}P_{yq}L_{\text{low}}\left(UB,q\right),$$ (8)

where **X***_s_* is the sequence data at site *s*. Using these formulas it is possible to update the values of *L*_upp_ and *L*_low_ once for each edge in the tree, and then perform branch-length optimization using only the values in *L*_upp_, *L*_low_, and the updated branch length. This substantially improves the efficiency of branch-length optimization, compared to performing a pruning algorithm across the entire tree. As shown similarly in Boussau *et al.* (2008), Equation 8 can be modified slightly to allow efficient relative-likelihood calculations for a given codon *q* at node *B*:

$$P\left({Χ}_{s}|G=g,\;B=q\right)=\sum_{y}L_{\text{upp}}\left(UB,y\right)P_{yq}L_{\text{low}}\left(UB,q\right).$$ (9)

Marginal ancestral state reconstruction can then be performed by calculating this value for each possible value of *q*, and selecting the codon with the highest relative probability. Alternatively, the most likely amino acid can be found by summing the marginal probabilities for all codons which code for that amino acid, and selecting the amino acid with the highest probability. For analyses in this article we compare marginal amino acid reconstructions. For reference, proofs of Equations 8 and 9, which are essentially the same as that in Boussau and Gouy (2006), are given in Supplemental Material, Figure S1 in File S1.

### Implementation

We implement this substitution model and likelihood calculation framework in IgPhyML, a program modified from the source code of codonPhyML (Gil *et al.* 2013). IgPhyML implements the rate matrix in Equation 1 and estimates the parameters *h^a^* using ML, together with the other model parameters. ML optimization is performed using the Broyden–Fletcher–Goldfarb–Shanno optimization algorithm that is implemented within codonPhyML (Gil *et al.* 2013). IgPhyML is available for download through https://github.com/kbhoehn/IgPhyML.

### bNAb lineage sequences and multiple sequence alignment

We applied our model to the heavy chain sequences of the three bNAb lineages analyzed in Wu *et al.* (2015). The lineage of greatest duration was VRC01, which was sampled over 15 years (Wu *et al.* 2015); followed by CAP256-VRC26 (hereafter VRC26), which was sampled over 4 years (Doria-Rose *et al.* 2014); and CH103, which was sampled over 3 years (Liao *et al.* 2013). Sequences from each bNAb lineage were downloaded from GenBank and translated into amino acids, aligned to their putative germline V-gene segment using IgBlast (Ye *et al.* 2013), and then retranslated back into codons. Putative germline segment assignments (V4-59*01 for CH103, V3-30*18 for VRC26, and V1-2*01 for VRC01) were obtained from bNAber (Eroshkin *et al.* 2013) and sequences were obtained from the IMGT V-Quest human reference set (Lefranc and Lefranc 2001). Because of considerable uncertainty in D and J germline assignments for each lineage, only the V segment was used. Insertions relative to the germline sequence were removed, so that all sequences within each lineage were aligned to the same germline sequence. Removing these insertions brought together 2 nt that are not actually adjacent, creating false motifs. To prevent this, the 3′ nt of the region joined together from the removal of the insertion was converted to an N. To keep results consistent among lineages, only nucleotide positions from the beginning of the first framework region (FWR1) to the end of FWR3 were used. Sampling dates of each sequence were extracted from the sequence identifier tags provided on GenBank. A total of 11 sequences were excluded from CH103 because this information was not available.

The three lineages showed substantial differences both in divergence and level of indels. While CH103 sequences showed no insertions relative to its germline sequence and VRC26 showed only two; VRC01 showed 394, many of which were the same length and occurred in the same position, and were thus likely inherited (*i.e.*, synapomorphic rather than homoplasic). In the final alignments for CH103, VRC26, and VRC01; 0.03, 0.07, and 0.5% of nucleotides were “N” characters, respectively. This state included gaps, germline-relative insertions, and ambiguous characters. Two sequences from VRC26 and two from VRC01 were removed due to difficulty in aligning them to the germline sequence, either because of high divergence or large indels. All three lineages showed high levels of root-tip divergence, with a mean of 27.6, 21.0, and 62.7% of codons differing between the root and tips for CH103, VRC26, and VRC01, respectively (Figure S2 in File S1).

### Hotspot model selection

Because we want to compare ancestral sequence reconstruction for specific nodes (see *Effects on ancestral state reconstruction*), and because previous studies have reported difficulties in finding optimal tree topologies with nonreversible models (Boussau and Gouy 2006), we chose to apply the HLP17 model to a fixed tree topology and the problem of coestimating topology will be addressed future work. For each data set, the tree topology used was that inferred using the standard M0 version of the GY94 model in codonPhyML, which was subsequently rerooted to place the germline sequence as the common ancestor of all tips.

Because WRC/GYW hotspots are generally cited as among the strongest hotspot motifs in SHM, and are generally thought to act in a strand-symmetric manner (Smith *et al.* 1996; Teng and Papavasiliou 2007; Yaari *et al.* 2013), the results of the symmetric WRC/GYW hotspot model fit are shown in detail in Table 1. By placing different constraints on the *h^a^* parameters, we tested 10 different hotspot models on the three bNAb lineages: CH103, VRC26, and VRC01. The specific constraints used to define each hotspot model and the results of model testing are shown in Table 3. Full results from each model fit are provided in Figure S3 in File S1. Importantly, all models that were compared using likelihood-ratio tests used the same, prespecified root sequence. The GY94/M0 model was specified as the HLP17 model with all *h^a^* fixed to zero.

**Table 1:** ML estimates of *h* and likelihood ratio tests for symmetric WRC/GYW model

Lineage	h^WRC¯/G¯YW	Log likelihood		2×LR	*P*-value
		*h*^WR^^C^^/^^G^^YW^ = MLE	*h*^WR^^C^^/^^G^^YW^ = 0		
CH103	1.86 (1.4, 2.4)	−14600.4	−14702	203.2	<1 × 10^−15^
VRC26	1.81 (1.5, 2.1)	−37238.5	−37516.4	555.8	<1 × 10^−15^
VRC01	2.03 (1.7, 2.4)	−43647.1	−43945.3	596.4	<1 × 10^−15^

Significance was determined using the likelihood ratio test under a chi-squared distribution with one degree of freedom. The 90% confidence intervals for $\hat{h}$ are shown in parentheses in the second column. MLE, ML estimate; LR, likelihood ratio.

Further, to ensure that the effects we observe are particular to the hotspot and coldspot motifs under investigation, we compared estimated *h* values for specific hotspot motifs to those obtained from all other possible trimer motifs with similar characteristics. Specifically, we generated all possible motifs and their reverse complements that (i) were 3 nt in length, (ii) contained two IUPAC letters standing for two possible nucleotides (R, Y, S, W, K, and M), and (iii) subsequently contained an unambiguous nucleotide (*i.e.*, A, C, G, or T). We then fitted the HLP17 model using each of these 144 motifs individually and compared how estimated *h* values for these motifs compared to the values for WRC/GYW and SYC/GRS. We repeated this process for dimer motifs but with the constraints that motifs (i) were 2 nt in length, (ii) contained one IUPAC letter standing for two possible nucleotides, and (iii) subsequently contained an unambiguous nucleotide. We fitted the HLP17 model to the same data using these 24-dimer motifs and compared them to the results from WA/TW motifs. Results from this analysis are shown in Figure S4 in File S1.

### Effectiveness of the mean-field approximation

We evaluated and validated the effectiveness of the HLP17 model by simulating data sets under different values of *h* and testing how accurately model parameters were inferred. For brevity, we considered only symmetric WRC/GYW hotspot motifs in this analysis (*h*^WR^^C^ *=* *h*^G^^YW^; hereafter in this section hereafter referred to as *h*). Because the HLP17 model is a mean-field approximation, it will not fully account for the context dependency of SHM. To measure the degree of this effect, we generated simulated data sets using a modified version of HLP17 that *does* fully account for the context dependence of adjacent codon sites. Specifically:

1. We estimated a ML phylogeny for each bNAb lineage data set using the standard GY94 model. During estimation we optimized *ω*, *κ*, *π_j_*, branch lengths, and the tree topology. The resulting ML tree was rerooted at the germline sequence with a branch length of zero.
2. For each value of *h* investigated (0, 1, 2, and 4), we simulated 20 alignments along each of these trees using a version of the model that fully accounts for context dependency. Specifically, in forward simulation, the 3′- and 5′-flanking codons of each site are known. This allowed us to create a **B** matrix for each site in each sequence with *b_ij_* equal to either 1 or 0, depending on whether or not the substitution was a hotspot mutation in a WRC/GYW motif. The process begins at the given root sequence and gives rise to a separate **B** and **Q** matrix at each site in the sequence. For each descendant branch, the matrix is multiplied by the branch length and then exponentiated to give the transition probability matrix; the next codon is selected randomly with respect to these probabilities. This process continues at each site to create two descendant sequences, then repeats for descendant nodes down the tree until all tips are filled. Simulations were undertaken using the estimated values of *ω*, *κ*, and π_j_ obtained in step 1 for the corresponding bNAb lineage data set.
3. For each of the replicates defined in step 2, we performed three different ML calculations: (i) *h* was optimized using ML (with $\hat{h}$ as the ML estimate of *h*), (ii) *h* was fixed to zero, and (iii) *h* was fixed to the true value used in simulation. These three scenarios enable us to test type-1 and type-2 error rates by determining whether $\hat{h}$ was significantly different to *h* or to zero, respectively. Statistical significance was determined using the chi-squared approximation to the likelihood-ratio statistic, as described above. In all calculations, the tree topology was fixed to that inferred in step 1.
4. For each data set and for each set of simulations under a particular value of *h*, we estimated $\hat{h}$ and then calculated the properties of this estimator as follows:
  - Bias in estimation: $\left(\mathrm{Mean}\left[\hat{h}\right]-h\right).$
  - Variance in estimation: $\mathrm{Variance}\left[\hat{h}\right].$
  - Type-1 error rate: The proportion of simulated data sets in which *h* was outside of the 95% confidence interval for $\hat{h}.$
  - Type-2 error rate: The proportion of simulated data sets in which *h* > 0, but failed to reject the null hypothesis (*h* = 0).

**Figure 2:**
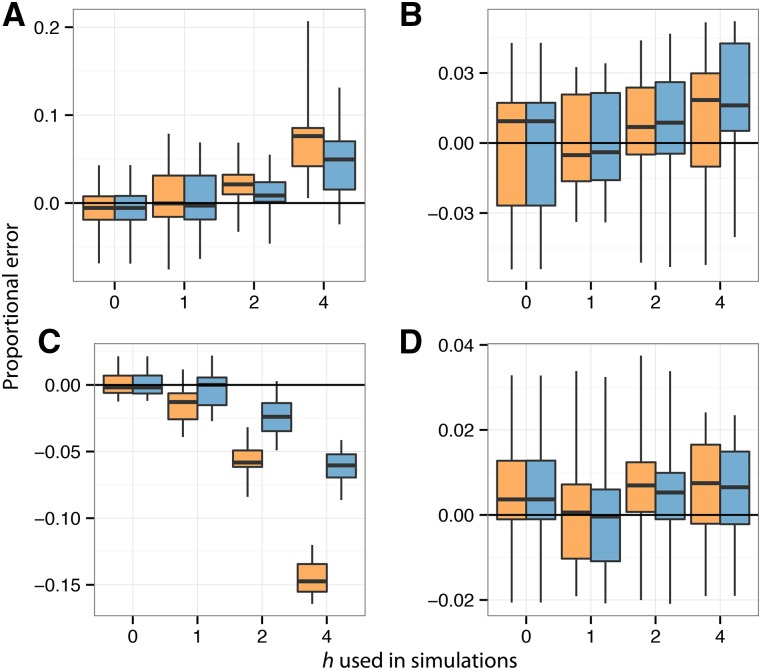
Proportional error in parameter estimation compared to true values for the VRC01 B-cell lineage, fully context-dependent simulations. Values of *ω*, *κ*, tree length, and ratio of internal to external branch lengths are shown in (A), (B), (C), and (D), respectively. Estimates obtained under the GY94 are in orange (*h* *=* 0) and estimates obtained under the HLP17 model are in blue (*h* estimated using ML). The edges and centers of box plots show the first, second, and third quartiles, while the whiskers show range. Similar results for B-cell lineages CH103 and VRC26 are shown in Figure S5 in File S1.

Biased mutation during SHM has been shown to give false signatures of natural selection using approaches that compare the expected number of replacement and silent mutations (Dunn-Walters and Spencer 1998). We hypothesized that the HLP17 model might partially reduce this bias. To test this, and to explore whether the HLP17 model improved estimation of other evolutionary parameters, we compared the percentage error under the HLP17 and GY94 models of estimates of (i) *ω*, (ii) *κ*, (iii) tree length (sum of all branch lengths), and (iv) the ratio of internal to external branch lengths. These results are provided in Figure 2 and Figure S5 in File S1.

Because *ω* and *κ* are estimated along with *h^a^*, it is possible that estimation of *h^a^* may interfere with the other parameters, or vice versa. To test this, we performed the fully context-dependent simulation procedure again for the CH103 lineage, but with different values of *h*^WR^^C^^/^^G^^YW^ (0, 1, 2, and 4) and *ω* (0.1, 0.5, and 1, 2). We then fit two models to each data set—the null model using the true values of *h*^WR^^C^^/^^G^^YW^ and *ω*, and the alternative model estimating both via ML—and compared them using the likelihood-ratio test. In these cases, the alternative model introduced two free parameters (*h* and *ω*) compared to the null model, so the likelihood-ratio test used a chi-square test with two degrees of freedom. The results of this simulation analysis are given in Figure S6 in File S1. To see whether this had any effect on hotspot model selection, we also fit an asymmetric WRC/GYW model for each simulated data set, to see if different values of *h* and *ω* led to more frequent rejection of the symmetric model. Further, we compared the values of $b_{ij}^{a},$ *ω*, and *κ* within the **Q** matrix of each best-fit HLP17 model, to see if any significant relationship existed between the parameters (Figure S6 in File S1).

The fact that bNAb lineages cannot be expected to be in equilibrium when they are sampled has interesting implications for the use of Markov substitution models. Typically, it is assumed that nucleotide or codon frequencies are at equilibrium at the time of sampling, and empirical codon frequencies are often used as estimates of equilibrium frequencies. In the case of long-lived B-cell lineages, however, sampled sequences are almost certainly not in equilibrium, making empirical codon frequencies inaccurate approximations for equilibrium frequencies. Because changes from SHM are not inherited through the germline, each BCR lineage is expected to begin out of sequence equilibrium, potentially converging to its equilibrium distribution as it evolves. To test how the use of empirical codon frequencies in such circumstances might affect estimation of *h*, we repeated the simulation procedure above using empirical equilibrium frequencies from each data set. These results are included in Figure S7 in File S1.

### Effects on ancestral state reconstruction

One of the key applications of molecular phylogenetics to BCR sequence data are the reconstruction of ancestral sequences within a B-cell lineage (Kepler 2013). For each bNAb lineage, we found the amino acid with the highest marginal probability at each site of each node using Equation 9. We performed this process for both the GY94 and best-fit HLP17 trees and parameters (*i.e.*, those obtained in the section *Hotspot model selection*). This allowed us to compare whether the HLP17 models showed any tangible difference in ancestral sequence reconstruction over GY94. However, because the ancestral sequences for the bNAb lineages are not known, we repeated this analysis on our lineages simulated under full context dependence, to see which model gave more accurate ancestral state reconstructions. We thus performed the same ancestral sequence reconstruction procedure for each simulation replicate, and for two of the models (*h* *=* ML, *h* *=* 0) described in step 3 above. These ancestral sequences were then used to compare the accuracy of reconstructions under the HLP17 model with those obtained using the GY94-type model (*h* = 0). In each simulation replicate, accuracy of ancestral sequence reconstruction was measured by calculating the mean number of amino acid differences between the predicted and true sequences at each node. These are shown in Figures S8 and S9 in File S1, respectively.

### Data availability

IgPhyML is available for download through https://github.com/kbhoehn/IgPhyML. Heavy chain sequences for CH103, VRC26, and VRC01 lineages are publicly available from the GenBank accession numbers listed in Liao *et al.* (2013), Doria-Rose *et al.* (2014), and Wu *et al.* (2015). The multiple sequence alignments used here, as well as source code for simulation and ancestral sequence reconstruction analyses, are included in File S2.

## Results

### A codon substitution model for phylogenies undergoing SHM

All three bNAb lineages showed a significant improvement in likelihood under the symmetric WRC/GYW HLP17 model compared to the GY94 model. The ML values of *h* for the three data sets were ${\hat{h}}^{\mathrm{WR}\underline{C}}$ = ${\hat{h}}^{\underline{G}YW}$ = 1.86, 1.81, and 2.03, for CH103, VRC26, and VRC01, respectively. In each case, the simpler GY94 model (all *h =* 0) could be rejected using the likelihood-ratio test (*P* < 0.0001 for all three lineages). These results are summarized in Table 1. These $\hat{h}$ values represent up to a threefold increase in the relative rate of change at hotspot locations (depending on the values of *b_ij_*).

The mean-field approximation used in this model did not dramatically affect parameter estimation when applied to data sets simulated under a fully context-dependent model, at least for the parameter space of the three empirical bNAb lineages (Table 2). Mean $\hat{h}$ values from simulations in which 0 ≤ *h*^WR^^C^^/^^G^^YW^ ≤ 2 were close to their true *h* values and exhibited low absolute bias and variability (maximum −0.204 and 0.028, respectively, when *h* = 2). Of these simulated data sets, 12.2% incorrectly rejected the correct parameter value (*i.e.*, they estimated an $\hat{h}$ significantly different from the true value of *h* used in the simulations). This is fairly close to the theoretical expectation under α = 0.05. Further, none of the data sets simulated with *h* > 0 failed to reject the null hypothesis that *h* = 0, demonstrating good statistical power. Bias generally increased if *h* was raised beyond that observed in the empirical bNAb linages. Performance was worse when *h* = 4, which resulted in a mean type-2 error of 0.62 and a mean bias of −0.35. This behavior is as expected because, as *h* increases, the mean-field approximation will become less accurate. We found that using empirical codon frequencies decreased the performance of *h* estimation; using empirical frequencies resulted in higher bias and type-2 error rates than using ML frequencies (Figure S7 in File S1). Discussion of why empirical codon frequencies are unlikely to be suitable for long-lived B-cell lineage phylogenies is provided in the *Materials and Methods* section.

**Table 2:** HLP17 performance under fully context-dependent simulations for symmetric WRC/GYW hotspots

Set	*h*	Mean $\hat{h}$	Bias	Variability	Type-1 error	Type-2 error
CH103	0.00	−0.012	−0.012	0.008	—	0.10
	1.00	1.091	0.091	0.018	0.00	0.05
	2.00	2.095	0.095	0.028	0.00	0.05
	4.00	4.146	0.146	0.093	0.00	0.05
VRC26	0.00	0.004	0.004	0.003	—	0.05
	1.00	0.965	−0.035	0.005	0.00	0.00
	2.00	1.841	−0.159	0.013	0.00	0.25
	4.00	3.501	−0.499	0.026	0.00	0.85
VRC01	0.00	−0.002	−0.002	0.002	—	0.05
	1.00	0.965	−0.035	0.008	0.00	0.05
	2.00	1.796	−0.204	0.022	0.00	0.50
	4.00	3.291	−0.709	0.049	0.00	0.95

Type-1 error rate shows the proportion of data sets that incorrectly failed to reject the null hypothesis of *h* = 0. Type-2 error rate shows the proportion of data sets that rejected the true value of *h* shown in the first column. Both hypothesis tests for type-1 and type-2 errors used an α value of 0.05. Importantly, data in these analyses were not simulations under HLP17, but a fully context-dependent variation of it.

Within the parameter space of the empirical data sets (0 ≤ *h*^WR^^C^^/^^G^^YW^ ≤ 2), there was no substantial difference in estimation of other model parameters compared to the standard GY94 model, except for the tree length parameter in some simulations (Figure 2 and Figure S5 in File S1). However, when this *h* is large (4, in these simulations), the GY94 model substantially underestimates tree length in each of the simulated lineages. In contrast, the HLP17 model, while not completely eliminating this effect, substantially reduced it. In simulations based on the long-lived VRC01 and CAP256 lineages in which this *h* = 4, the GY94 model overestimated the *ω* parameter; this bias was not obvious in simulations based on the CH103 lineage, which was sampled for a shorter duration. The HLP17 model performed slightly better in *ω* estimation under these circumstances. Interestingly, for data set CH103, HLP17 showed a higher error in *κ* for *h* = 4.

The distribution of *b_ij_* weights for individual motifs, and to a lesser extent combined symmetric motifs, were noticeably different for synonymous and nonsynonymous substitutions (Figure S6, E and F, in File S1). To see how this relationship may affect our results, we simulated data sets under the symmetric WRC/GYW model with different values of *h* and *ω*, and fit both symmetric and asymmetric WRC/GYW models to each data set. Interestingly, under the symmetric model we found no consistent pattern of rejecting the true values of *h* and *ω* as these parameters increased. The asymmetric model did, however, show an increase in incorrectly rejecting the symmetric model as *h* and *ω* increased. This appears to have been driven by bias in *h*^G^^YW^, which is overestimated, and to a lesser extent *h*^WR^^C^, which was underestimated, as *h* and *ω* increased (Figure S6B in File S1). Interestingly, there was no such consistent bias in *h*^WR^^C^^/^^G^^YW^ under the symmetric WRC/GYW motif, and neither symmetric nor asymmetric models showed a robust bias in *ω* (Figure S6C in File S1).

### Hotspot model selection

All hotspot motif models tested gave a significantly higher likelihood than the standard GY94 model when applied to the CH103, VRC26, and VRC01 B-cell lineages. Likelihoods were considerably higher for asymmetric models. Using a likelihood-ratio test, the asymmetric WRC/GYW model showed a significantly better fit than the corresponding nested symmetric model (*P* = 4.6 × 10^−15^, 6.3 × 10^−5^, and 5.2 × 10^−3^ for lineages CH103, VRC26, and VRC01, respectively). Similarly, the asymmetric WA/TW model fitted the data better than its symmetric counterpart (*P* < 1 × 10^−15^ for all three lineages). Allowing different hotspot motifs to have different *h* values also resulted in significantly higher likelihoods than using a uniform value of *h* for all hotspots (*P* < 1 × 10^−14^ for all three lineages). Interestingly, VRC26 and VRC01 showed a significantly higher likelihood under asymmetric SYC/GRS coldspot motifs (*P* = 2.0 × 10^−13^ and 4.2 × 10^−3^), but CH103 did not (*P* = 0.53). This difference was also reflected in the best-fit (lowest AIC) model for each lineage. For VRC26 and VRC01, the best-fit model was the “free coldspots and hotspots” model, in which all motifs and their reverse complements are given separate *h* values. However, for CH103 the best-fit model was the “symmetric coldspots, asymmetric hotspots” model, in which each hotspot and its reverse complement are given separate *h* values, but coldspots remain symmetric.

Our randomization analysis confirmed that the hotspots we have selected do indeed evolve differently to similarly constrained random motifs. Known hotspots were hotter, and known coldspots were colder, than nearly all such random motifs. Further, random motifs that showed better fit than known hotspots were similar to known hotspots, differing only in the site furthest from the mutable position. WRC/GYW motifs exhibited a larger value of *h*, and a higher likelihood, than any other trimer motif analyzed. Further, SYC/GRS motifs resulted in *h* values that were <142 of the 143 other trimer motifs tested (only KYC/GRM showed a lower *h*, and was the only other trimer motif that showed a higher likelihood and negative *h*). WA/TW motifs showed a higher *h* value than all other dimer motifs analyzed, and a higher likelihood than 21 of the 23 other dimer motifs (only RC/GY and YC/GR showed higher likelihoods). These results are shown in Figure S4 in File S1.

### Ancestral state reconstruction

In fully context-dependent simulations, we also found that the HLP17 model provided an accuracy of ancestral state reconstructions that was similar to the GY94 model where *h* < 4, and that HLP17 noticeably improved accuracy at *h* = 4 (Figure S9 in File S1). While true ancestral sequences are not available for the three empirical bNAb lineages, we did observe differences between ancestral sequences reconstructed using the HLP17 and GY94 models. For each lineage, we compared the two models by calculating the mean number of amino acid differences between the predicted ancestral sequences at all internal nodes of each tree. Performing this ancestral state reconstruction on each of the three bNAb lineages showed a mean of 0.04-, 0.1-, and 0.09-aa sequence difference across all internal nodes, with a maximum difference of 3-, 2-, and 4-aa differences in a single node for CH103, VC26, and VRC01, respectively. Differences were somewhat more concentrated in the basal third of the phylogeny. Typically, we would expect the uncertainty in ancestral state reconstruction to increase as we move from the tree tips toward the root; however, B-cell lineages are unusual in that the root sequence is also known as it corresponds to the germline sequence.

## Discussion

Molecular phylogenetics has already been used in a variety of applications in the study of BCR genetic diversity and the molecular evolution of B-cell lineages (Kepler 2013; Sok *et al.* 2013; Kepler *et al.* 2014). However, the process of SHM is known to occur in ways that violate fundamental assumptions of most phylogenetic substitution models. Here, we demonstrate that failing to account for this has tangible effects on phylogenetic inference from sets of sequences from long-lived bNAb lineages. We develop and implement a new codon substitution model (HLP17) that, while only an approximation, is capable of mitigating these effects.

Perhaps the most salient difference between standard substitution models and the biology of SHM is the context dependency of mutation in BCRs. This biased mutation process at hotspot motifs; for which a variety of empirical models have been developed to characterize the process at di-, tri-, penta-, and heptamer levels (Smith *et al.* 1996; Yaari *et al.* 2013; Elhanati *et al.* 2015); has long been known to give a false signature of selection in BCRs (Dunn-Walters and Spencer 1998). This effect was observed in some of our simulations (Figure 2 and Figure S5 in File S1), as a failure to account for the increased rate of substitution at hotspot motifs led to overestimation of the *ω* (*d*_N_/*d*_S_) parameter. However, these simulations used an *h* value of 4, which was outside of the range of what we observed for empirical bNAb lineages.

Other approaches have been taken to study the effect of context-dependent mutation in phylogenetic substitution models. Many have focused on modeling the substantially increased mutability of CpG motifs (Hwang and Green 2004; Lunter and Hein 2004). These approaches are attempts to account for the full context dependency of CpG hypermutation, and require significantly more complex models. In the case of SHM in BCRs, the increased mutability of BCR hotspot mutations (approximately threefold) is not as great as CpG motifs (∼18-fold; Lunter and Hein 2004), so a simpler, approximate approach is still effective (Table 2). The mean-field approximation has also been used previously, but in a reversible codon model, to take into account di- and trinucleotide substitutions (Whelan and Goldman 2004).

The HLP17 codon substitution model detailed here is a relatively straightforward modification of the widely used M0 submodel of the GY94 model. Although it requires special techniques for efficient likelihood computation and ancestral state reconstruction (see *Materials and Methods*), we expect it to be not only usable, but statistically preferable to the GY94 model when applied to any BCR data set whose diversity may have been shaped by SHM. Further, the HLP17 model does not rely on an empirical model to incorporate the effect of biased mutation, but instead attempts to explicitly model the context-dependent mutational process by estimating the parameter(s) *h^a^* directly from the data. We note, however, that the HLP17 model is a mean-field approximation and does not capture the full context of motif-driven evolution. Therefore we do not expect it to fully disentangle interactions between selection and biased mutation, and estimated values of *ω* should be interpreted carefully. Importantly, empirical analyses on bNAb lineages performed here were using tree topologies that were optimal under GY94, rather than HLP17. This is expected to make the estimation of each *h* conservative in these analyses, but it is not clear how the optimal topology of the HLP17 model will differ from that under GY94.

Our model selection results suggest that different hotspot motifs have highly variable effects on sequence evolution in B-cell lineages. It is generally thought that increased mutation in WRC/GYW motifs (or the tetramer motifs WRCY/RGYW) reflect the action of activation-induced cytidine deaminase targeting, while in WA/TW motifs it is the result of error-prone polymerase repair (Teng and Papavasiliou 2007). Consistent with these separate mechanisms, WRC/GYW motifs have generally been found to be strand symmetric, but WA/TW motifs are strand biased, with WA mutating at a higher rate than TW (Bransteitter *et al.* 2004; Spencer and Dunn-Walters 2005; Teng and Papavasiliou 2007). It is interesting, then, that all three lineages tested here show a significantly better fit for asymmetric *vs.* symmetric WRC/GYW (Figure S3 in File S1 and Table 3). However, we believe these results do not necessarily conflict with previous findings on the targeted nature of SHM, and can be explained by other factors. Our simulation analyses using the CH103 lineage show that increasing values of *h* and *ω* can lead to an artificially high *h*^G^^YW^, an artificially low *h*^WR^^C^, and an increased rate of incorrectly rejecting the symmetric WRC/GYW model in favor of the asymmetric version (Figure S6, A and B, in File S1). This may be due to how, in the **Q** matrix, *ω* interacts differently between *b*^WR^^C^ and *b*^G^^YW^ (Figure S6E in File S1). Importantly, this bias appears to be limited to the *h* parameter in the asymmetric model, as the same simulations showed no consistent bias in *ω* for the symmetric model, and only a weak bias for the asymmetric model (Figure S6C in File S1). Further, increasing *ω* in these simulations and analyzing them using the symmetric WRC/GYW model did not reveal bias in *h*^WR^^C^^/^^G^^YW^ (Figure S6B in File S1), or an increased rejection of the true values of *ω* and *h*^WR^^C^^/^^G^^YW^ (Figure S6D in File S1). Thus, while the symmetric WRC/GYW model likely does fit better due to the increased mutability of WRC/GYW motifs (see also Figure S4 in File S1), the biological significance of its rejection in favor of the asymmetric WRC/GYW model should be interpreted with caution.

**Table 3:** Hotspot model selection

Model name	Constraint/optimization of each *h^a^*						*P*-values from LR tests			
	*h*^WR^^C^	*h*^G^^YW^	*h*^W^^A^	*h*^T^^W^	*h*^SY^^C^	*h*^G^^RS^		CH103	VRC26	VRC01
Symmetric WRC/GYW*^a^*	ML	*h*^WR^^C^	0	0	0	0		4.6 × 10^−15^	6.3 × 10^−05^	5.2 × 10^−03^
Asymmetric WRC/GYW	ML	ML	0	0	0	0				
Symmetric WA/TW*^a^*	0	0	ML	*h*^W^^A^	0	0		<1 × 10^−15^	<1 × 10^−15^	<1 × 10^−15^
Asymmetric WA/TW	0	0	ML	ML	0	0				
Symmetric SYC/GRS*^a^*	0	0	0	0	ML	*h*^SY^^C^		0.53	2.0 × 10^−13^	4.2 × 10^−03^
Asymmetric SYC/GRS	0	0	0	0	ML	ML				
Uniform hotspots*^a^*	ML	*h*^WR^^C^	*h*^WR^^C^	*h*^WR^^C^	0	0		4.2 × 10^−15^	<1 × 10^−15^	<1 × 10^−15^
Hierarchical hotspots	ML	*h*^WR^^C^	ML	*h*^W^^A^	0	0				
SCAH*^a^*	ML	ML	ML	ML	ML	*h*^SY^^C^		0.65	1.2 × 10^−06^	9.1 × 10^−04^
FCH	ML	ML	ML	ML	ML	ML				

Models of hotspot hierarchy (degree of mutability) and symmetry, specified by placing constraints on how different values of *h* are optimized. Columns 2–7 show how the parameter *h^a^* is obtained for a particular model. A value of 0 indicates that *h* is fixed at zero, ML indicates that a parameter is optimized by ML, and *h^a^* indicates that *h* parameter is equal to another value of *h*. For instance, in *Symmetric WRC/GYW*, *h*^G^^YW^ is equal to its reverse complement *h*^WR^^C^, which is ML optimized. However, in *Asymmetric WRC/GYW*, both are ML optimized. Rows 8–10 show *P*-values obtained from likelihood-ratio tests of each of these nested hotspot models for the bNAb lineage specified in each column. Parameters, log likelihood, and AIC of each fit are shown in Figure S3 in File S1. LR, likelihood ratio; SCAH, symmetric coldspots, asymmetric hotspots; FCH, free coldspots and hotspots.

a Each of these models is nested with the model immediately below it by one free parameter, allowing hypothesis testing using a likelihood-ratio test.

Another common assumption in phylogenetic analysis is that the codons or nucleotides sampled for analysis are at their equilibrium frequencies throughout the phylogeny. Because codon frequencies in the B cell’s germline ancestor may be different from the equilibrium frequencies of the somatic substitution process, the somatic evolutionary process may begin out of equilibrium. As a result, mean codon frequencies may change through time within a B-cell lineage, until convergence. This phenomenon is not related to the nontime reversibility of our model; it will occur even under a time-reversible model. We dealt with this problem in two ways. The first is in only using codon frequencies in the **Q** matrix, rather than also using them at the root during likelihood calculations. This effectively separates the codon frequencies from the germline substitution process (the root sequence) from the somatic substitution process (the **Q** matrix). For simplicity, we estimated the codon frequencies of the somatic substitution process by ML within the model. This provided an improvement, both in ML and in parameter estimation, over using empirical codon frequencies (Figure S7 in File S1). However, it is not yet clear if this is the most efficient or the most effective way of dealing with the problem of sequences that have not converged to their equilibrium distribution. While ML optimization finds the best-fitting single set of codon frequency values (under the CF3x4 model), in reality codon frequencies may change over the course of the phylogeny, and a model that accounts for that would likely be more appropriate. While it should be possible to calculate the expected change in codon frequencies over a defined branch length given a nonreversible substitution model, we leave that problem for future analyses.

The decay of hotspot motifs in bNAb lineages may have important implications for our understanding of host–virus coevolution. More specifically, the loss of hotspot motifs may lead to a decrease in sequence mutability, and therefore a decline in overall rate of evolution over time for a given lineage (Sheng *et al.* 2016). This hypothesis has several interesting implications. If the slowdown in mutation rate over time, arising from hotspot decay, is an intrinsic property of activated B-cell lineages, then BCR sequence divergence from a germline ancestor (and thus affinity maturation) may be intrinsically constrained. Consequently, while BCR lineages may be able to rapidly evolve binding affinity and coevolve with pathogens for an initial period after activation, over longer periods of time the ratio of the rate of BCR-sequence change to pathogen-sequence change may decline. We hypothesize that in extreme cases the rates of BCR evolution within a lineage may eventually fail to keep up with the rapid evolution of chronically infecting viruses, such as HIV-1, due to the exhaustion of available BCR hotspot motifs. The notion that biased mutation will lead to decreased mutability and evolutionary rate was explored recently by Sheng *et al.* (2016). They concluded that the observed mutation rate decreases in bNAb lineages was most likely due to a shift from positive to purifying selection, although the loss of hotspot motifs may also play a role and the issue is not yet fully resolved.

We have implemented this model in the software package IgPhyML, a modified version of codonPhyML (Gil *et al.* 2013). This program can perform all of the substitution model analyses performed here. Source code is available at https://github.com/kbhoehn/IgPhyML.

## Supplementary Material

Supplemental material is available online at www.genetics.org/lookup/suppl/doi:10.1534/genetics.116.196303/-/DC1.
