# Supplementary material for: A Phylogenetic Codon Substitution Model for Antibody Lineages
Source: Genetics. 2017 Mar 16;206(1):417–27. doi: 10.1534/genetics.116.196303 (PMC5419485; doi:10.1534/genetics.116.196303)
Supplement: Supplementary file 1 [file 417FileS1.pdf]

Supplemental Information
A phylogenetic codon substitution model for antibody lineages

Kenneth B. Hoehn
Gerton Lunter
Oliver G. Pybus

March 16, 2017

Contents

1 Likelihood computation and ancestral state reconstruction 2

2 Summary statistics of bNAb sequence data 5

3 Parameters from different motif models 6

4 Analysis with random motifs 7

5 Parameter results from fully context dependent simulation 8

6 Analysis of interaction between h and ω 10

7 Simulation analysis using empirical codon frequencies 14

8 Ancestral reconstruction results in bNAb lineages 15

9 Ancestral reconstruction results from simulations 16

1 Likelihood computation and ancestral state reconstruction

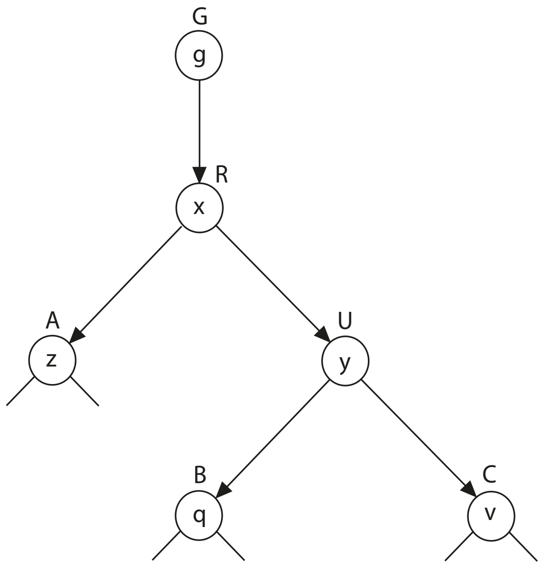

Evolutionary model reproduced from main text Figure 1

Supplemental Figure 1: An algorithm for efficient likelihood computation and marginal ancestral sequence reconstruction with a non-reversible model on sequences with a known common ancestor. See Figure 1 (reproduced above). All sequences descend from given germline node G, which has sequence g. Note that this known ancestor G is not necessarily the most recent common ancestor of the lineage, which is node R and sequence x. See similarities to tree and terminology in Boussau and Gouy (2006).

P_{gx} = Transition probability from g to x along branch GR.

$$L_{low}(UC, v) = \begin{cases} 1 & \text{v = character at tip} \\ 0 & \text{otherwise} \end{cases} \tag{1}$$

$$L_{low}(RU, y) = \sum_q P_{yq} L_{low}(UB, q) \sum_v P_{yv} L_{low}(UC, v) \tag{2}$$

$$L_{upp}(RU, x) = P_{gx} \sum_z P_{xz} L_{low}(RA, z) \tag{3}$$

$$L_{upp}(UB, y) = \sum_x P_{xy} L_{upp}(RU, x) \sum_v P_{yv} L_{low}(UC, v) \tag{4}$$

Supplemental Figure 1a: Calculating likelihood at each node This is effectively the same proof from (Boussau and Gouy 2006) but with P_{gx} in place of equilibrium frequencies.

Full likelihood P(data, given germline character g):

$$L_s = P(X|G = g) = \sum_x P_{gx} \sum_z P_{xz} L_{low}(RA, z) \sum_y P_{xy} \sum_q P_{yq} L_{low}(UB, q) \sum_v P_{yv} L_{low}(UC, v) \quad (5)$$

Likelihood from a germline edge:

$$\begin{aligned} L_{s,GR} = P(X|G = g) &= \sum_x P_{gx} L_{low}(GR, x) \\ &= \sum_x P_{gx} \sum_z P_{xz} L_{low}(RA, z) \sum_y P_{xy} L_{low}(RU, v) \\ &= \sum_x P_{gx} \sum_z P_{xz} L_{low}(RA, z) \sum_y P_{xy} \sum_q P_{yq} L_{low}(UB, q) \sum_v P_{yv} L_{low}(UC, v) \\ &= L_s \end{aligned} \quad (6)$$

Likelihood from a root edge:

$$\begin{aligned} L_{s,RU} = P(X|G = g) &= \sum_x L_{upp}(RU, x) \sum_y P_{xy} L_{low}(RU, y) \\ &= \sum_x P_{gx} \sum_z P_{xz} L_{low}(RA, z) \sum_y P_{xy} \sum_q P_{yq} L_{low}(UB, q) \sum_v P_{yv} L_{low}(UC, v) \\ &= L_s \end{aligned} \quad (7)$$

Likelihood from a non-root edge:

$$\begin{aligned} L_{s,UB} = P(X|G = g) &= \sum_y L_{upp}(UB, y) \sum_q P_{yq} L_{low}(UB, q) \\ &= \sum_y \sum_x P_{xy} L_{upp}(RU, x) \sum_v P_{yv} L_{low}(UC, v) \sum_q P_{yq} L_{low}(UB, q) \\ &= \sum_x L_{upp}(RU, x) \sum_y P_{xy} \sum_v P_{yv} L_{low}(UC, v) \sum_q P_{yq} L_{low}(UB, q) \\ &= \sum_x L_{upp}(RU, x) \sum_y P_{xy} L_{low}(RU, y) \\ &= L_{s,RU} = L_s \end{aligned} \quad (8)$$

Supplemental Figure 1b: Marginal ancestral state reconstruction The essential operation of marginal ancestral state reconstruction, is, for a given node N, find the character n which maximizes $P(X|G=g,N=n)$.

Reconstruction at node X:

$$P(X|G=g)=\sum_x P_{gx}\sum_z P_{xz}L_{low}(RA,z)\sum_y P_{xy}\sum_q P_{yq}L_{low}(UB,q)\sum_v P_{yv}L_{low}(UC,v) \tag{9}$$

$$P(X|G=g,X=x)=P_{gx}\sum_z P_{xz}L_{low}(RA,z)\sum_y P_{xy}\sum_q P_{yq}L_{low}(UB,q)\sum_v P_{yv}L_{low}(UC,v) \tag{10}$$

Then beginning at branch GR:

$$\begin{aligned} P(X|G=g,X=x)_{GR} &= P_{gx}L_{low}(GR,x) \\ &= P_{gx}\sum_z P_{xz}L_{low}(RA,z)\sum_y P_{xy}L_{low}(RU,v) \\ &= P_{gx}\sum_z P_{xz}L_{low}(RA,z)\sum_y P_{xy}\sum_q P_{yq}L_{low}(UB,q)\sum_v P_{yv}L_{low}(UC,v) \\ &= P(X|G=g,X=x) \end{aligned} \tag{11}$$

Reconstruction at node B:

$$P(X|G=g,B=q)=\sum_x P_{gx}\sum_z P_{xz}L_{low}(RA,z)\sum_y P_{xy}P_{yq}L_{low}(UB,q)\sum_v P_{yv}L_{low}(UC,v) \tag{12}$$

Then beginning at branch UB:

$$\begin{aligned} P(X|G=g,B=q)_{UB} &= \sum_y L_{upp}(UB,y)P_{yq}L_{low}(UB,q) \\ &= \sum_y\sum_x P_{xy}L_{upp}(RU,x)\sum_v P_{yv}L_{low}(UC,v)P_{yq}L_{low}(UB,q) \\ &= \sum_y\sum_x P_{xy}P_{gx}\sum_z P_{xz}L_{low}(RA,z)\sum_v P_{yv}L_{low}(UC,v)P_{yq}L_{low}(UB,q) \\ &= \sum_x P_{gx}\sum_z P_{xz}L_{low}(RA,z)\sum_y P_{xy}P_{yq}L_{low}(UB,q)\sum_v P_{yv}L_{low}(UC,v) \\ &= P(X|G=g,B=q) \end{aligned} \tag{13}$$

2 Summary statistics of bNAb sequence data

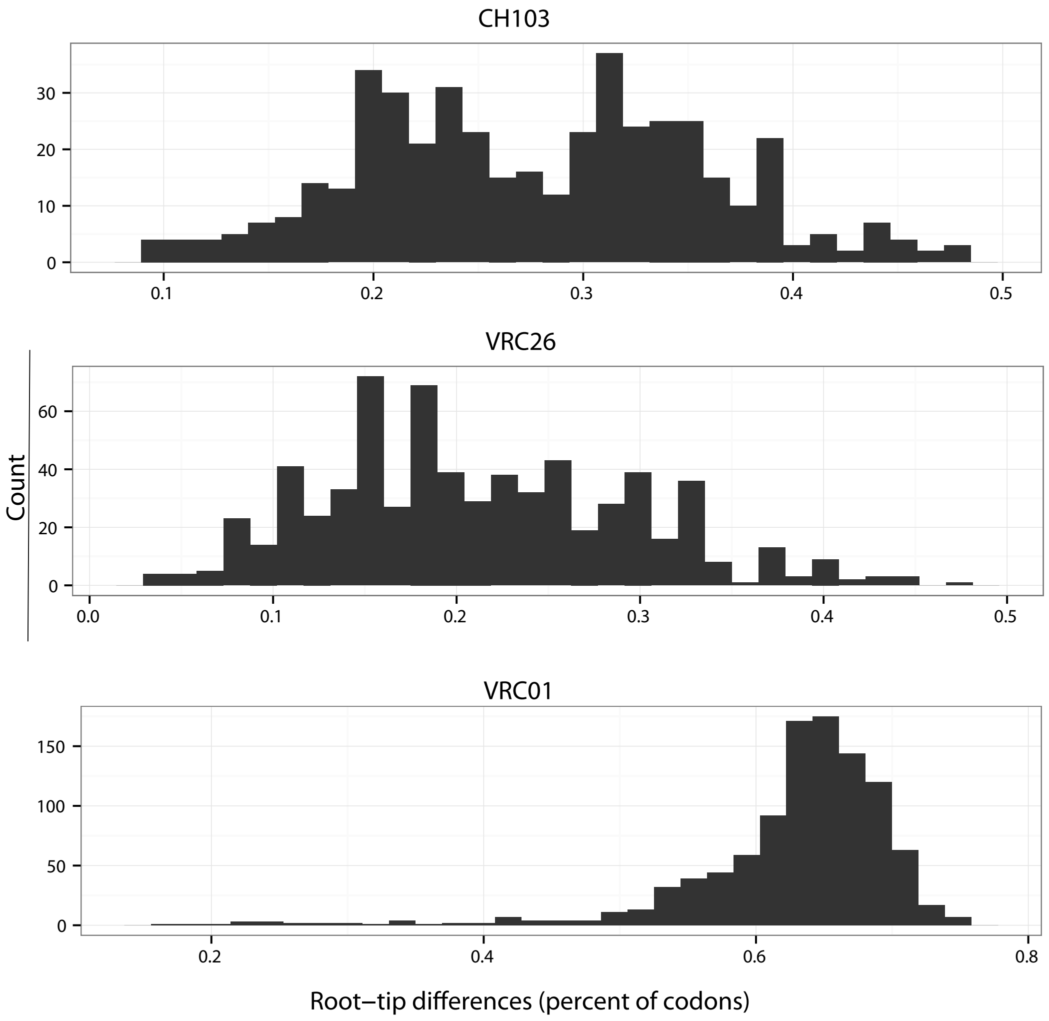

Supplemental Figure 2: Distribution of pairwise codon differences from each tip sequences to the germline sequence of each lineage.

3 Parameters from different motif models

Set	Hotspot model	κ	ω	h^{WRC}	h^{GYW}	h^{WA}	h^{TW}	h^{SYC}	h^{GRS}	Log L.hood	AIC
CH103	Symm. WRC/GYW*	1.91	0.43	1.86	1.86	0	0	0	0	-14600.4	29230.8
	Asymm. WRC/GYW	1.98	0.42	1.19	3.50	0	0	0	0	-14569.7	29171.4
	Symm. WA/TW*	1.89	0.41	0	0	0.64	0.64	0	0	-14667.7	29365.4
	Asymm. WA/TW	1.84	0.37	0	0	1.54	-0.18	0	0	-14605	29242
	Symm. SYC/GRS*	1.88	0.41	0	0	0	0	-0.57	-0.57	-14658.3	29346.6
	Asymm. SYC/GRS	1.88	0.41	0	0	0	0	-0.59	-0.54	-14658.1	29348.2
	Uniform hotspots*	1.87	0.39	2.31	2.31	2.31	2.31	0	0	-14513	29056
	Hierarchical hotspots	1.88	0.40	3.87	3.87	1.96	1.96	0	0	-14482.2	28996.4
	SCAH*	1.90	0.34	2.31	5.84	3.23	0.59	-0.21	-0.21	-14388.8	28815.6
	FCH	1.90	0.35	2.30	5.86	3.22	0.59	-0.19	-0.24	-14388.7	28817.4
VRC26	Symm. WRC/GYW*	1.87	0.56	1.81	1.81	0	0	0	0	-37238.5	74507
	Asymm. WRC/GYW	1.87	0.56	2.16	1.48	0	0	0	0	-37230.5	74493
	Symm. WA/TW*	1.89	0.54	0	0	0.74	0.74	0	0	-37386.3	74802.6
	Asymm. WA/TW	1.88	0.52	0	0	1.13	0.29	0	0	-37344.4	74720.8
	Symm. SYC/GRS*	1.88	0.56	0	0	0	0	-0.62	-0.62	-37385.4	74800.8
	Asymm. SYC/GRS	1.89	0.57	0	0	0	0	-0.38	-0.78	-37358.4	74748.8
	Uniform hotspots*	1.85	0.50	2.55	2.55	2.55	2.55	0	0	-36915.8	73861.6
	Hierarchical hotspots	1.85	0.51	4.09	4.09	2.18	2.18	0	0	-36837.4	73706.8
	SCAH*	1.83	0.49	4.37	3.34	2.62	1.33	-0.14	-0.14	-36794	73626
	FCH	1.83	0.50	4.19	3.39	2.56	1.29	0.15	-0.39	-36782.2	73604.4
VRC01	Symm. WRC/GYW*	2.22	0.33	2.03	2.03	0	0	0	0	-43647.1	87324.2
	Asymm. WRC/GYW	2.23	0.33	1.80	2.31	0	0	0	0	-43643.2	87318.4
	Symm. WA/TW*	2.22	0.33	0	0	0.38	0.38	0	0	-43899.2	87828.4
	Asymm. WA/TW	2.21	0.32	0	0	0.80	-0.09	0	0	-43823.8	87679.6
	Symm. SYC/GRS*	2.19	0.33	0	0	0	0	-0.69	-0.69	-43742.5	87515
	Asymm. SYC/GRS	2.19	0.33	0	0	0	0	-0.74	-0.61	-43738.4	87508.8
	Uniform hotspots*	2.21	0.33	1.49	1.49	1.49	1.49	0	0	-43596.8	87223.6
	Hierarchical hotspots	2.21	0.33	3.51	3.51	1.19	1.19	0	0	-43436.9	86905.8
	SCAH*	2.19	0.32	2.42	3.01	1.32	0.30	-0.48	-0.48	-43327.1	86692.2
	FCH	2.18	0.31	2.50	2.96	1.35	0.32	-0.57	-0.34	-43321.6	86683.2

Supplemental Figure 3: ML parameters, log-likelihood, and AIC values for each model fit on each bNAb lineage. See Table 4 for model descriptions. SCAH = Symmetric coldspots, asymmetric hotspots. FCH = Free coldspots and hotspots.

4 Analysis with random motifs

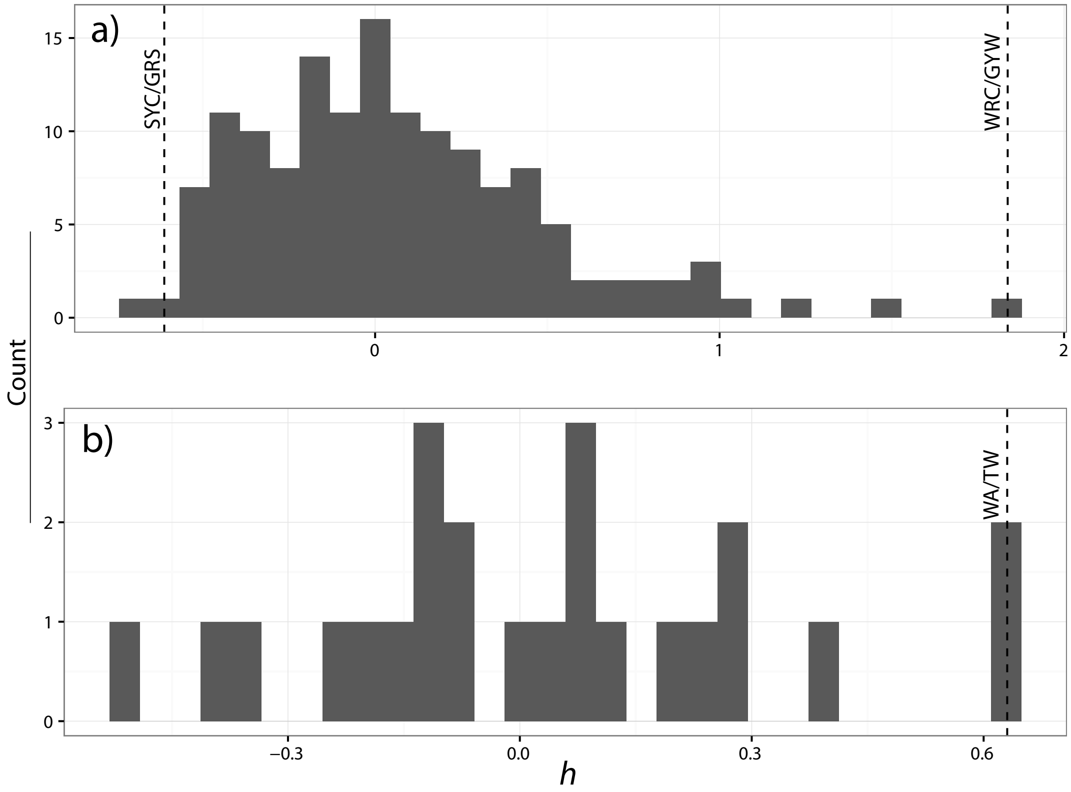

Supplemental Figure 4: Distribution of h values for all possible motifs fulfilling the following criteria: a) trimers which contained two IUPAC letters standing for two possible nucleotides (R, Y, S, W, K, and M), which were followed by an unambiguous nucleotide (A, C, G, or T), and b) dimer which contained one IUPAC letters standing for two possible nucleotides, which was followed by an unambiguous nucleotide. We then fit symmetric HLP17 models using each of these motifs and their reverse complements, with all other h parameters set to zero.

5 Parameter results from fully context dependent simulation

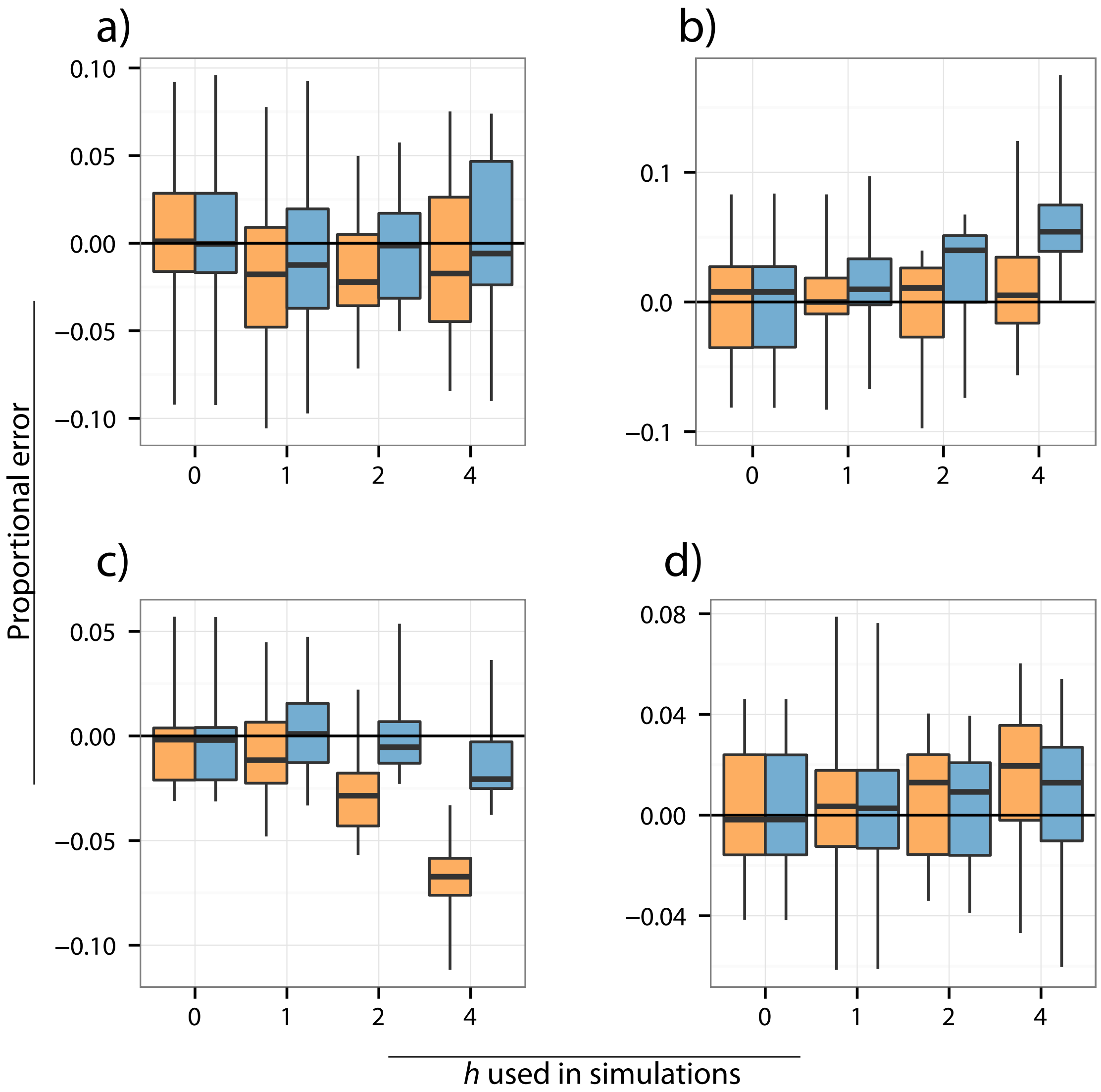

Supplemental Figure 5a: Proportional error in parameter estimation compared to true values for the CH103 B cell lineage fully context dependent simulations. Values of ω, κ, tree length, and ratio of internal to external branch lengths are shown in panels a), b), c), and d), respectively. Estimates obtained under the GY94 are in orange (h=0) and estimates obtained under the HLP17 model are in blue (h estimated using maximum likelihood). The edges and centres of boxplots show the 1st, 2nd, and 3rd quartiles, while the whiskers show range.

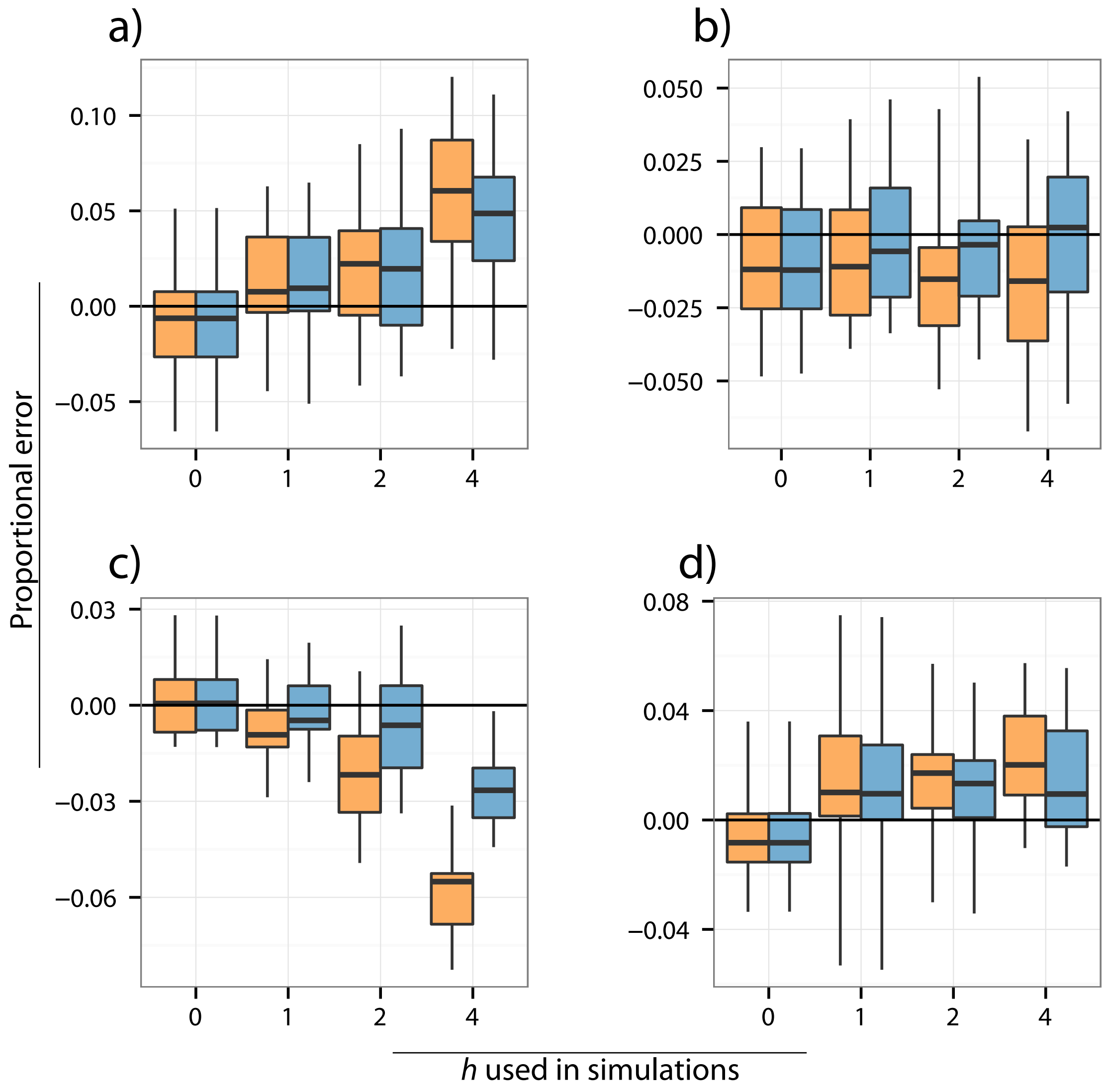

Supplemental Figure 5b: Proportional error in parameter estimation compared to true values for the VRC26 B cell lineage fully context dependent simulations. Values of ω, κ, tree length, and ratio of internal to external branch lengths are shown in panels a), b), c), and d), respectively. Estimates obtained under the GY94 are in orange (h=0) and estimates obtained under the HLP17 model are in blue (h estimated using maximum likelihood). The edges and centres of boxplots show the 1st, 2nd, and 3rd quartiles, while the whiskers show range.

6 Analysis of interaction between h and ω

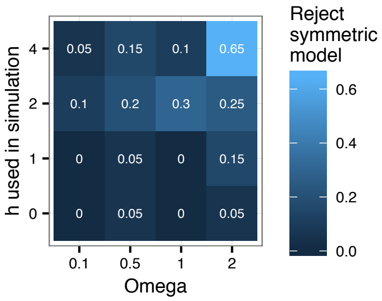

Supplemental Figure 6a: Proportion of fully context-dependent simulated data sets that incorrectly reject the true symmetric WRC/GYW motif model. Note that this generally increases as both h and ω increase.

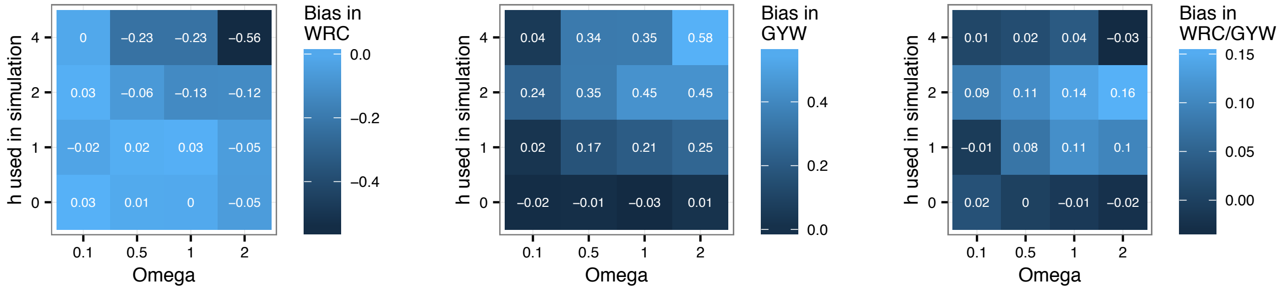

(a) (b) (c)

Supplemental Figure 6b: Bias in h from data sets simulated using different values of h and ω under a fully context-dependent symmetric WRC/GYW model. Note that in the asymmetric model h^{WRC} and h^{GYW} are biased in opposite directions as h and ω increase, while in the symmetric model h^{WRC} shows no such consistent bias. Note that these values are absolute bias, not proportional error. This would be obtained by dividing each value in each cell by the true h used in simulation (left axis).
a) Bias in the value of h^{WRC} estimated from fitting an asymmetric WRC/GYW model.
b) Bias in the value of h^{GYW} estimated from fitting an asymmetric WRC/GYW model.
c) Bias in the value of $h^{WRC/GYW}$ estimated from fitting a symmetric WRC/GYW model.

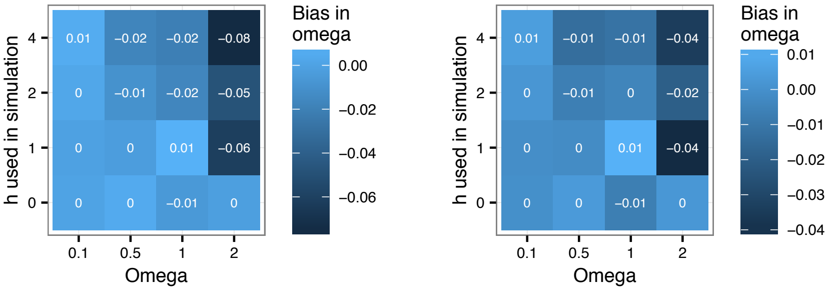

(a) (b)

Supplemental Figure 6c: Bias in ω from data sets simulated using different values of h and ω under a fully context-dependent symmetric WRC/GYW model. Note that, while a trend exists in the asymmetric model, the bias is ultimately very small.

a) Bias in the value of ω estimated from fitting an asymmetric WRC/GYW model.

b) Bias in the value of ω estimated from fitting a symmetric WRC/GYW model.

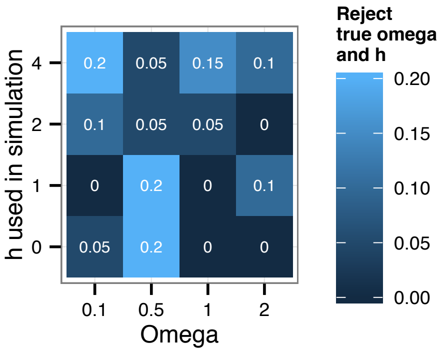

Supplemental Figure 6d: Proportion of fully context-dependent simulated data sets simulated under the symmetric WRC/GYW model that incorrectly reject the true values of h and ω.

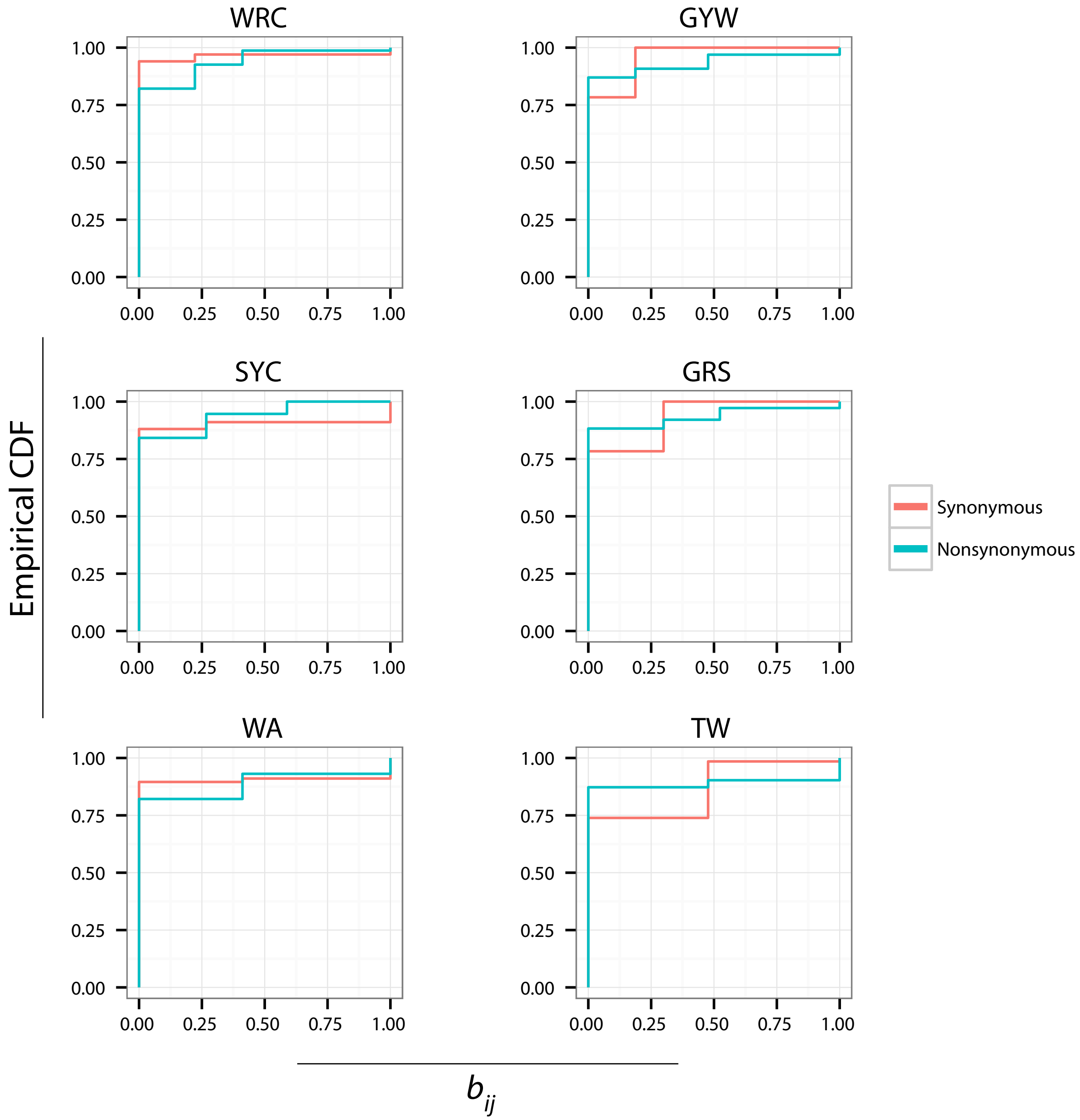

Supplemental Figure 6e: Cumulative distribution of b_{ij} parameters among synonymous vs non-synonymous substitutions in the Q matrix formed from different motifs individually using the ML codon frequency results from CH103. Results are nearly identical in the other two lineages.

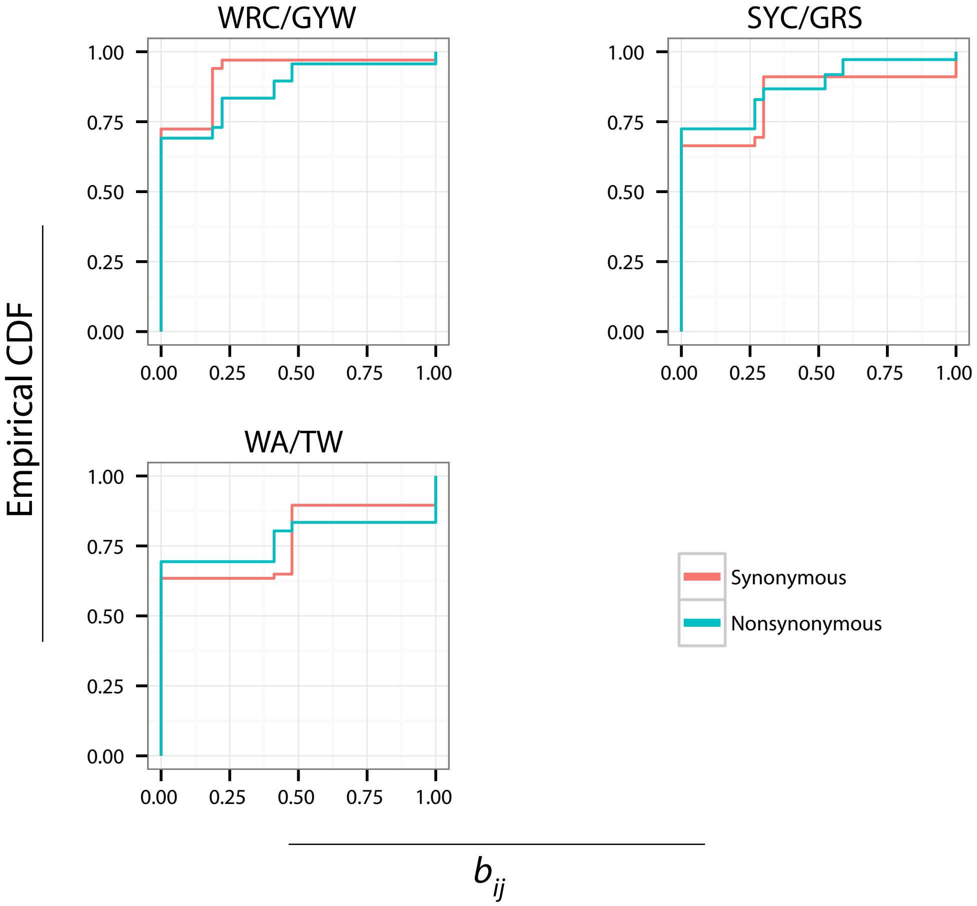

Supplemental Figure 6f: Cumulative distribution of b_{ij} parameters among synonymous vs non-synonymous substitutions in the Q matrix formed from different motifs together and symmetrically using the ML codon frequency results from CH103. Results are nearly identical in the other two lineages.

7 Simulation analysis using empirical codon frequencies

Set	h	MLE h	Bias	Variability	Type 1 error	Type 2 error
CH103	0	-0.042	-0.042	0.009	-	0.25
	1	0.829	-0.171	0.021	0	0.35
	2	1.546	-0.454	0.020	0	0.85
	4	2.935	-1.065	0.062	0	1
VRC26	0	-0.011	-0.011	0.003	-	0
	1	0.894	-0.106	0.006	0	0.35
	2	1.719	-0.281	0.008	0	0.85
	4	3.126	-0.874	0.042	0	1
VRC01	0	0.032	0.032	0.006	-	0.25
	1	0.758	-0.242	0.012	0	0.8
	2	1.270	-0.730	0.031	0	1
	4	2.167	-1.833	0.028	0	1

Supplemental Figure 7: Effects of empirical equilibrium frequencies on h estimation. Type 1 error rate shows the proportion of data sets that incorrectly failed to reject the null hypothesis of $h = 0$. Type 2 error rate shows the proportion of data sets that rejected the true value of h shown in the first column. Both of these hypothesis tests used an alpha value of 0.05. See Methods for explanation of how summary statistics were calculated.

8 Ancestral reconstruction results in bNAb lineages

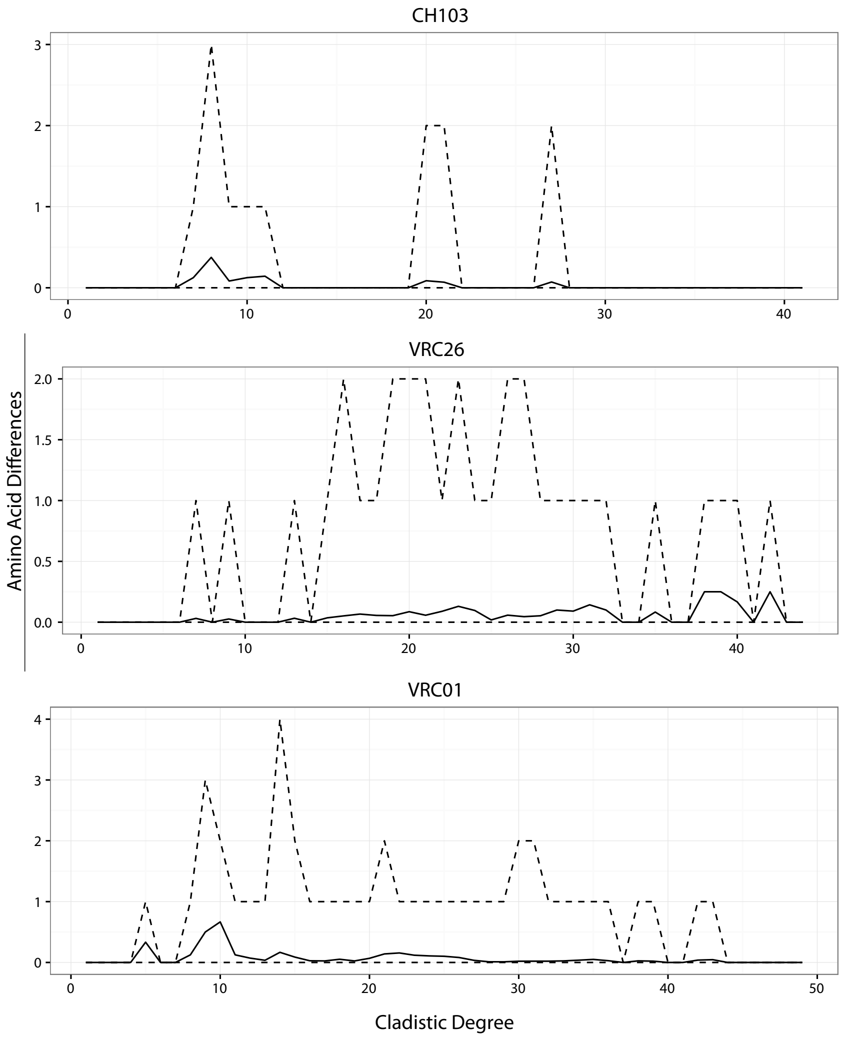

Supplemental Figure 8: Differences between predicted ancestors for the best fit HLP17 model (Supplemental Figure 3) and the GY94 model for three bNAb lineages by cladistics degree (nodes from root sequence). Solid line shows the mean difference for each node, dashed lines show the minimum and maximum values for each degree.

9 Ancestral reconstruction results from simulations

Set	h	Reconstructed sites per dataset	Mean incorrect amino acids per dataset: h=MLE	h=0	Improvement for h=MLE
CH103	0	36207	25.05	25.2	0.15
	1		25.65	25.4	-0.25
	2		25.1	25.85	0.75
	4		24.95	28.6	3.65
VRC26	0	64992	62.55	62.7	0.15
	1		64.2	64.05	-0.15
	2		67.05	68	0.95
	4		67.35	72.9	5.55
VRC01	0	98880	88.9	88.75	-0.15
	1		85.25	85.45	0.2
	2		87.25	91.45	4.2
	4		89.85	99.3	9.45

Supplemental Figure 9: Accuracy of ancestral state reconstruction in simulation analyses. The mean number of incorrectly reconstructed amino acids per data set are shown for the h=MLE and $h = 0$ models, for each set of h parameters used in simulations.
